# Nonlinear statistical iterative reconstruction for propagation-based phase-contrast tomography

**DOI:** 10.1063/1.4990387

**Published:** 2018-01-23

**Authors:** Lorenz Hehn, Kaye Morgan, Pidassa Bidola, Wolfgang Noichl, Regine Gradl, Martin Dierolf, Peter B. Noël, Franz Pfeiffer

**Affiliations:** 1 Chair of Biomedical Physics, Department of Physics and Munich School of Bioengineering, Technical University of Munich, 85748 Garching, Germany; 2 Department of Diagnostic and Interventional Radiology, Klinikum rechts der Isar, Technical University of Munich, 81675 Munich, Germany; 3 Institute for Advanced Study, Technical University of Munich, 85748 Garching, Germany; 4 School of Physics and Astronomy, Monash University, Clayton, VIC 3800, Australia

## Abstract

Propagation-based phase-contrast tomography has become a valuable tool for visualization of three-dimensional biological samples, due to its high sensitivity and its potential in providing increased contrast between materials with similar absorption properties. We present a statistical iterative reconstruction algorithm for this imaging technique in the near-field regime. Under the assumption of a single material, the propagation of the x-ray wavefield—relying on the transport-of-intensity equation—is made an integral part of the tomographic reconstruction problem. With a statistical approach acting directly on the measured intensities, we find an unconstrained nonlinear optimization formulation whose solution yields the three-dimensional distribution of the sample. This formulation not only omits the intermediate step of retrieving the projected thicknesses but also takes the statistical properties of the measurements into account and incorporates prior knowledge about the sample in the form of regularization techniques. We show some advantages of this integrated approach compared to two-step approaches on data obtained using a commercially available x-ray micro-tomography system. In particular, we address one of the most considerable challenges of the imaging technique, namely, the artifacts arising from samples containing highly absorbing features. With the use of statistical weights in our noise model, we can account for these materials and recover features in the vicinity of the highly absorbing features that are lost in the conventional two-step approaches. In addition, the statistical modeling of our reconstruction approach will prove particularly beneficial in the ongoing transition of this imaging technique from synchrotron facilities to laboratory setups.

## I. INTRODUCTION

As opposed to conventional x-ray absorption imaging, which relies solely on the attenuation of the x-rays in matter, phase-contrast imaging (PCI) is sensitive to x-ray phase shifts. This technique is becoming more and more important in laboratory and preclinical studies, yielding distinct advantages for the visualization of weakly absorbing details that are common in biological and medical samples. By extending PCI to computed tomography (CT),^1^ it has become a valuable tool for three-dimensional visualization of thick and complex samples due to its high sensitivity and its potential in providing increased contrast between materials of similar absorption properties.^2,3^

The most intuitive way to obtain phase contrast is via propagation-based methods,^4,5^ which rely on the fact that when a coherent wavefront traverses a sample and propagates sufficiently far to a detector, the phase shifts induced by the sample lead to distinct variations in the measured intensity through interference effects. In contrast to grating-based methods^6,7^ or analyzer-based methods,^8,9^ propagation-based methods do not require additional apparatus in the beam-path. In addition, propagation-based PCI does not place strict requirements on the monochromaticity of the source.^10^

Iterative algorithms have already been successfully applied to various techniques for propagation-based phase-contrast tomography. For instance, for propagation distances in the Fresnel regime, the phase information can be retrieved from a single propagation distance using iterative algorithms that cycle between the detection plane and the object plane applying several constraints.^11^ This approach has also been extended to tomography.^12^ Other iterative methods rely on the linearization of the image formation process using various forms of the free-space contrast transfer function.^13,14^ By acquiring multiple images at different distances for each tomographic angle, it has been shown that the phase information can accurately be recovered for multi-material^15^ and heterogeneous objects.^16^ However, these multi-distance techniques have only been performed at synchrotron facilities in the literature to date, perhaps due to the sensitivity of existing multi-distance algorithms to noise,^17^ access to a reduced region of the contrast transfer function (due to finite source size),^18^ or difficulties in image alignment in the presence of magnification. Recent work has looked at the problem of image alignment in the context of CT,^19^ with the promise of new phase retrieval algorithms for multiple distances. Therefore, at laboratory sources, it is more common to use direct methods that recover phase information from propagation-based PCI measurements from only a single projection. Many analytical phase-retrieval algorithms have been developed for this purpose, each imposing different restrictions onto the sample.^20,21^ An early method, derived by Bronnikov, assumes a pure-phase object and is therefore only valid for weakly absorbing samples.^22^ The most widely used algorithm from Paganin *et al.* assumes that the sample consists of only one material.^23^ This leads to artifacts in regions where this assumption fails. The reconstruction of multi-material samples can be performed using Beltran's generalization of Paganin's phase retrieval. However, in the case of more than two materials, the multiple reconstructions that come from each pair of materials must be spliced together manually,^24,25^ or retrieval should use a two-step method in the case of three materials.^26^ Alternatively, if images are collected at multiple distances, an iterative multi-distance reconstruction algorithm can be used.^15,16^

With the growing importance of statistical iterative reconstruction (SIR) approaches for conventional x-ray absorption CT,^27^ we believe that our approach will also prove to be beneficial for propagation-based phase-contrast CT. Thereby, we model the whole nonlinear image formation process using the assumptions of the phase-retrieval approach of Paganin *et al.* suitable for laboratory sources. For CT, we directly integrate the phase retrieval into a statistical approach for tomographic reconstruction that acts on the measured intensities, tackling some of the current challenges related to this imaging technique. SIR techniques are known to be very flexible and can incorporate noise statistics, sophisticated geometries, physical models, and prior knowledge.^28^ Furthermore, these approaches are capable of reducing the required dose significantly while maintaining the image quality.^29–33^

## II. IMAGE FORMATION FORMULATION

In the following, we outline the mathematical formulations and assumptions around the image formation process that builds the foundations of our iterative algorithms. In general, the image formation process can be divided into two parts: the interaction of the incoming x-rays with the sample and the free space propagation to the detector. First of all, an expression is derived that recovers the wavefield in the contact-plane directly behind the sample. Second, the relationship between the measured intensity at the detector-plane and the wavefield in the contact-plane is explained.

In order to be able to describe the interactions of the x-ray wavefield with the sample independent of propagation, it is assumed that the sample is sufficiently thin or scatters sufficiently weakly to neglect any propagation effects within the sample.^34^ This is known as the projection approximation.^11^ Like in the phase-retrieval algorithm of Paganin *et al.*,^23^ the assumption is made that our sample consists of only one material. This has the huge advantage of coupling the intensity and phase properties of the wavefield and thus halving the number of parameters necessary to obtain the wavefield in the contact plane. By assuming a monochromatic forward-propagating scalar wave, the wavefield behind the sample can be decomposed into its intensity *I*(**r**_⊥_) and phase *ϕ*(**r**_⊥_) by

$$\psi (r_{⊥})=\sqrt{I(r_{⊥})}e^{i\phi (r_{⊥})},$$ (1)

where **r**_⊥_ describes the coordinates orthogonal to the propagation direction. Knowing the material's specific attenuation coefficient *μ*, the real part of the deviation of the complex refractive index from unity *δ*, as well as the wave number *k* (dependent solely on the energy of the x-rays), we can recover the intensity and the phase from the trace *T*(**r**_⊥_) that corresponds to the projected thickness of the sample along the x-ray direction, namely

$$I(r_{⊥})=e^{-\mu T(r_{⊥})},$$ (2)

$$\phi (r_{⊥})=-k\delta T(r_{⊥}).$$ (3)

Thereby, the trace *T*(**r**_⊥_) is the projection denoted by the operator P of the three-dimensional distribution of the sample *t*(**r**) along the x-ray paths

$$T(r_{⊥})=Pt(r).$$ (4)

Evidently, in the case of tomography, a distinct wavefield is generated under each tomographic angle.

The origin of the subsequent derivations is the transport-of-intensity equation (TIE),^35^ which describes the evolution of the x-ray wavefield intensity due to propagation. It can be derived by inserting Eq. (1) in the paraxial Helmholtz equation and isolating the imaginary part.^11^ The TIE has the following form:

$$\nabla_{⊥}\cdot \left(I(r_{⊥},z)\nabla_{⊥}\phi (r_{⊥},z)\right)=-k\frac{\partial }{\partial z}I(r_{⊥},z).$$ (5)

Evaluating the derivative along *z*, assuming that the contact- and detector-planes are sufficiently close to each other such that the intensity evolves over this distance in a way that is linear in *z*,^11^ yields

$$\tilde{I}(r_{⊥})=I(r_{⊥})-\frac{z}{k}\nabla_{⊥}\cdot \left(I(r_{⊥})\nabla_{⊥}\phi (r_{⊥})\right),$$ (6)

where we denote the intensity part of the wavefunction in the contact plane as *I*(**r**_⊥_) and accordingly its phase as *ϕ*(**r**_⊥_). The intensity in the detector plane is denoted by $\tilde{I}(r_{⊥})$. If we again assume a homogeneous object, we can use Eqs. (2) and (3) to make the intensity distribution in the detector-plane $\tilde{I}(r_{⊥})$ solely dependent on the trace *T*(**r**_⊥_) of the object

$$\tilde{I}(r_{⊥})=e^{-\mu T(r_{⊥})}+z\delta \nabla_{⊥}\cdot \left(e^{-\mu T(r_{⊥})}\nabla_{⊥}T(r_{⊥})\right).$$ (7)

The algorithm of Paganin *et al.*^23^ (PAG) recovers the trace *T*(**r**_⊥_) analytically from Eq. (7) and has the following form:

$$T(r_{⊥})=-\frac{1}{\mu }\ln\left(F_{⊥}^{-1}\left(\frac{F_{⊥}\left(\tilde{I}(r_{⊥})\right)}{z\frac{\delta }{\mu }k_{⊥}^{2}+1}\right)\right),$$ (8)

where **k**_⊥_ are the corresponding spatial frequencies of **r**_⊥_ and $F_{⊥}$ denotes the two-dimensional Fourier transform.

## III. DERIVATION OF THE ITERATIVE ALGORITHMS

**FIG. 1.**
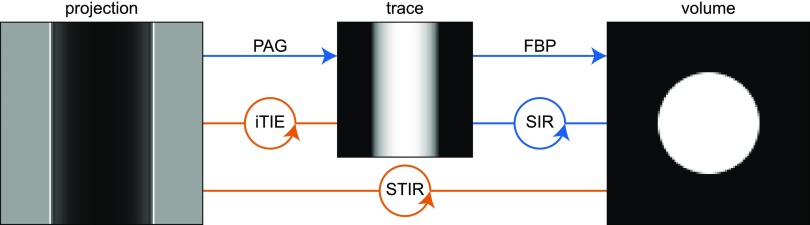
Scheme of various reconstruction techniques. Usually, the trace is recovered from the projections by the reconstruction algorithm of Paganin *et al.* (PAG). As an intermediate step, we compare this approach with a more flexible iterative algorithm (iTIE) to achieve this goal. The volume is recovered by filtered back-projection (FBP) or more advanced statistical iterative reconstruction techniques (SIR). Finally, we introduce a transport-of-intensity based iterative reconstruction algorithm (STIR) that recovers the volume directly from the measured projections. Those algorithms shown in orange are described for the first time in this paper.

Analogous to the image formation, reconstructing the spatial distribution of the material usually requires two steps as illustrated in Fig. 1. The classic reconstruction process uses Eq. (8) (PAG, as the phase-retrieval component of the reconstruction) to retrieve the traces from the measured intensities obtained by illuminating the object under different angles. The three-dimensional volume is then reconstructed by a filtered back-projection algorithm (FBP, the computed tomography component of the reconstruction) from these traces. However, for the reconstruction of the volume from the traces, one could also use more sophisticated SIR algorithms that make use of prior knowledge via regularizing techniques as well as additional weights on the traces.

As an intermediate step, we establish an iterative TIE-based algorithm (iTIE) that recovers the trace from the measured intensity in the near-field and includes the possibilities of statistical weighting of the measurements and regularization techniques on the traces. As our main finding, we present a statistical TIE-based iterative reconstruction approach (STIR) for reconstructing the distribution of the material from tomographic measurements acquired in the near-field regime. Our algorithm accounts for the noise statistics of the measurements, recovers the three-dimensional distribution of the sample without the intermediate step of recovering the traces, and makes use of regularization techniques. Figure 1 illustrates this together with the common reconstruction approaches discussed previously.

### A. iTIE

Our first algorithm is based on the propagation of the x-rays. We further manipulate Eq. (7) by neglecting the cross-term $z\delta \left[\nabla_{⊥}\;\text{exp}\;\{-\mu T(r_{⊥})\}\right]\cdot \nabla_{⊥}T(r_{⊥})$, where the squared brackets indicate the scope of the ∇_⊥_ operator, by assuming that at a particular position in the wavefield, the product of the intensity gradient and the phase gradient is comparably small, and end up with the following simplified model:

$$\tilde{I}(r_{⊥})=e^{-\mu T(r_{⊥})}\left(1+z\delta \nabla_{⊥}^{2}T(r_{⊥})\right),$$ (9)

which is the basis of our iterative reconstruction algorithm.

As a first step to obtain our forward-model, which relates the quantity we are interested in to our measurements, Eq. (9) is discretized, merging the coordinates orthogonal to the propagation direction into one dimension

$${\tilde{I}}_{i}=e^{-\mu T_{i}}\left(1+z\delta \sum_{k}b_{ik}T_{k}\right),$$ (10)

where the Laplace operator is rewritten as a matrix *b_ik_* using the five-point stencil finite-difference method. From now on, the indices *i*, *k* run over the total number of pixels in the corresponding planes.

Finally, we can establish a noise model that accounts for the statistical properties of our measurements. For simplicity, we restrict ourselves to a Gaussian noise model although other noise models could be used as well. However, a pure Poisson model does not hold for CCD cameras, as commonly used in x-ray phase-contrast imaging. In addition, a Poisson model is well approximated by a Gaussian model for the number of counts observed in any reasonable imaging situation. We establish a cost-function of the following form:

$$L=\frac{1}{2}\sum_{i}w_{i}{\left({\tilde{I}}_{i}-{\tilde{y}}_{i}\right)}^{2}+\lambda R\prime .$$ (11)

The actual measurements in the detector plane are denoted by ${\tilde{y}}_{i}$. The first part of this equation is denoted as the data-term. Due to our noise model, it penalizes the quadratic differences of our forward model to the actual measurements. To account for the fact that different data-points vary in their significance, it is reasonable to set the statistical weights *w_i_* to the inverse variance of the measured data. In practice, the variance is directly estimated from the measured intensity. The second part of Eq. (11) is referred to as the regularization-term. The regularizer R′ acts on the traces and incorporates prior knowledge on the sample. Usually, the regularizer penalizes solutions where noise is present. The coupling parameter between the data-term and the regularization-term is denoted by *λ* and has to be chosen accordingly.

The goal is to minimize the cost-function L in order to find the optimal trace $T^{*}$ coinciding well with the measurements according to the noise statistics and prior knowledge of the regularizer. This can be written as

$$T^{*}=\underset{T}{\mathrm{arg min}}L,$$ (12)

and solved using iterative methods for solving unconstrained nonlinear optimization problems. Due to the fact that this approach minimizes the simplified TIE iteratively, we refer to it as iTIE.

If as a special case we assume a non-absorbing object *μ_i_* = 0 and recover the phase from its trace, namely, *ϕ_i_* = −*kδT_i_*, we are left with a forward model whose analytical solution coincides with the phase-retrieval algorithm of Bronnikov^22^ but has the benefits of a statistical iterative approach as discussed above.

### B. STIR

In the case of propagation-based phase-contrast tomography, the aim is not to recover the traces under each tomographic angle but to directly recover the three-dimensional distribution of the material. In order to account for the projection process, we discretize Eq. (4) and extend it to multiple angles

$$T_{i}^{\theta }=\sum_{j}a_{ij}^{\theta }t_{j},$$ (13)

where *j* now runs over all voxels of the three-dimensional volume and *θ* indicates the respective projection angle. For simplicity, avoiding too much notation overhead, the three spatial dimensions of the volume are again merged into a single dimension. The first part of the image formation process is a linear operation and can therefore be described by a matrix multiplication, denoted by $a_{ij}^{\theta }$.

As we want to omit any intermediate steps, we insert Eq. (13) into our discrete model for propagation, Eq. (10). Thus, our forward model describes the expected intensity distribution on the detector for all angles depending solely on the distribution of the material

$${\tilde{I}}_{i}^{\theta }=e^{-\mu \sum_{j}a_{ij}^{\theta }t_{j}}\left(1+z\delta \sum_{k}b_{ik}\sum_{j}a_{kj}^{\theta }t_{j}\right).$$ (14)

Analogous to Eq. (11), we establish a cost function

$$L=\frac{1}{2}\sum_{\theta }\sum_{i}w_{i}^{\theta }{\left({\tilde{I}}_{i}^{\theta }-{\tilde{y}}_{i}^{\theta }\right)}^{2}+\lambda R,$$ (15)

which now runs over all pixels and all tomographic angles. The measurements under different tomographic angles are denoted by ${\tilde{y}}_{i}^{\theta }$. The regularizer R now acts on the volume.

Now, we minimize Eq. (15) by an iterative method for solving unconstrained nonlinear optimization problems to end up with the optimal distribution of the material $t^{*}$ coinciding with our measurements according to their noise statistics and prior knowledge, written as

$$t^{*}=\underset{t}{\mathrm{arg min}}L.$$ (16)

We refer to this algorithm as STIR because it describes the SIR approach to CT reconstruction, integrated with the TIE iterative trace retrieval from propagation-based PCI.

Again, as a special case, if we assume a pure absorption object *δ* = 0 or equivalently the detector being located in the contact-plane *z* = 0 and recover the attenuation coefficients *μ_i_* = *μt_i_*, we find an optimization problem, which acts on the intensities rather than on the line-integrals, as it is common for absorption tomography. Our model therefore circumvents the error introduced by estimating the line-integrals, due to Jensen's inequality, as discussed in Ref. 27.

## IV. IMPLEMENTATION

In the following, the details of the implementation of the derived algorithms are presented along with the conventional tomographic reconstruction approaches and regularization techniques used for comparison.

### A. Tomographic reconstruction

Recovering the three-dimensional distribution of the material from its traces according to Eq. (13) is analogous to the reconstruction of the absorption coefficients from line-integrals in absorption tomography. It can be performed by FBP or SIR techniques.

Due to the cone-beam geometry of the x-ray micro-tomography system, the FBP technique of our choice is the algorithm of Feldkamp *et al.*^36^ For SIR, using a Gaussian noise model, we can establish a cost-function of the following form:

$$L=\frac{1}{2}\sum_{\theta }\sum_{i}w_{i}^{\theta }{\left(T_{i}^{\theta }-y_{i}^{\theta }\right)}^{2}+\lambda R,$$ (17)

to recover the spatial distribution of the material. In this case, $y_{i}^{\theta }$ are the traces under different angles recovered by PAG.

### B. Regularization

In the following, we use total-variation regularization^37^ that relies on the assumption that our volume is piecewise constant and only considers the discrepancies between neighboring voxels, which can be written as

$$R=\frac{1}{2}\sum_{j}\sum_{n\in N_{j}}\frac{1}{d_{jn}}{\left\|\frac{x_{j}-x_{n}}{d_{jn}}\right\|}_{1}.$$ (18)

In this formulation, *j* runs again over all voxels and *n* over the next neighbors of *j*. The neighborhood $N_{j}$ holds therefore 26 voxels. The different distances to the neighbors are taken into account by the additional factors $d_{jn}\in \{1,\sqrt{2},\sqrt{3}\}$.

### C. Implementation details

All algorithms are implemented on a heterogeneous computer consisting of multiple central processing units (CPUs) and graphical processing units (GPUs). The implementation of the cone-beam projection operation, as described in Eq. (13), and its transpose operation for calculating the gradients of the cost-functions are described in Ref. 38. Like the projection operations, regularization is also implemented on multiple GPUs using OpenCL. The remaining calculations of the models as in Eqs. (10) and (14) are implemented in python on multiple CPUs. An optimization routine implemented as a python wrapper to the Limited-memory Broyden-Fletcher-Goldfarb-Shanno routine (L-BFGS) described in Ref. 39 is used. Thereby, the cost-functions as described in Eqs. (11) and (15) and the corresponding gradients have to be calculated.

## V. RESULTS

In the following, we validate the derived algorithms and compare them with common reconstruction approaches using datasets obtained at an x-ray micro-tomography system (Xradia 500, Carl Zeiss, USA). The energy assumed in the following examples is 13 keV, as motivated in Ref. 40. The values for *δ* and *μ* for the different measurements are extracted from the xraylib library^41^ according to the material, density, and energy.

### A. Validation of iTIE

First of all, we validate the propagation part of our model by showing that the iTIE algorithm, as a first step towards a fully iterative algorithm for tomography, coincides with the findings of the PAG algorithm. Therefore, we use a projection of a 250 *μ*m wide teflon plate with an effective propagation distance of *z* = 8.57 mm and an effective pixel size in the object plane of 0.964 *μ*m. The acquisition of the projection of the teflon plate is discussed in Ref. 40. The distances are 10 mm from the source to the sample and 60 mm between the object and the detector. Due to the magnification effects, this leads to the stated effective propagation distance.

**FIG. 2.**
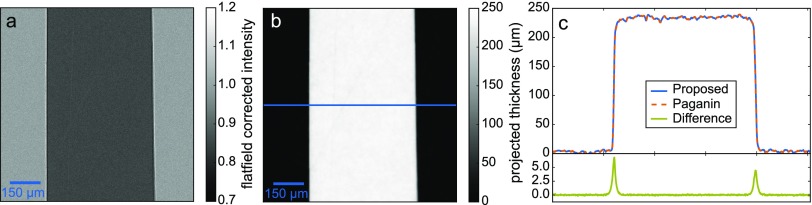
Validation of the iterative phase-retrieval algorithms on a projection. The flat-field corrected intensity distribution of the 250 *μ*m Teflon plate at the detector plane is depicted in (a). The trace retrieved with the iterative phase-retrieval algorithm iTIE is shown in (b). This algorithm is compared with the PAG algorithm in (c), where the line profiles of the center row of the traces for both algorithms can be seen in the upper part, while the lower part illustrates the differences between the two methods.

As depicted in Fig. 2(a), in addition to the attenuation of the teflon plate, edge-enhancement effects are visible at the transition from teflon to air. These regions hold information about the phase-shifting properties of the sample, which play a significant role in recovering the trace. To make the iTIE algorithm comparable with the PAG algorithm, we omitted any additional weighting *w_i_* = 1 and regularization *λ* = 0.

Minimization according to Eq. (12) for the iTIE algorithm leads to the trace depicted in Fig. 2(b), which coincides well with the properties of the sample. As the initial guess, a plane of zeros is used. Figure 2(c) compares the result with the PAG algorithm for the central row. In blue the profile of the according row of Fig. 2(b) is plotted. Using the PAG algorithm as in Eq. (8) yields the dashed line-plot in orange for the central row. In green the difference of the two line profiles is shown.

The two methods coincide except for small deviations at the edges of the teflon plate, making this approach applicable for quantitative phase retrieval, as for instance performed in analyzing the air volume in lungs.^42^ The small deviation can be explained by the neglection of the cross-terms in Eq. (9). In principle, these additional terms could be included in the iterative framework. Furthermore, solving the equation in the spatial domain avoids wrapping artifacts at the borders due to the fact that the borders in the spatial domain can be mirrored or clamped to the edges. Although the analytical algorithm is much faster, it lacks possibilities for weighting the projection, for instance, according to its statistical properties, masking of features and regularization techniques on the projected thicknesses.

### B. Benefits of STIR

**FIG. 3.**
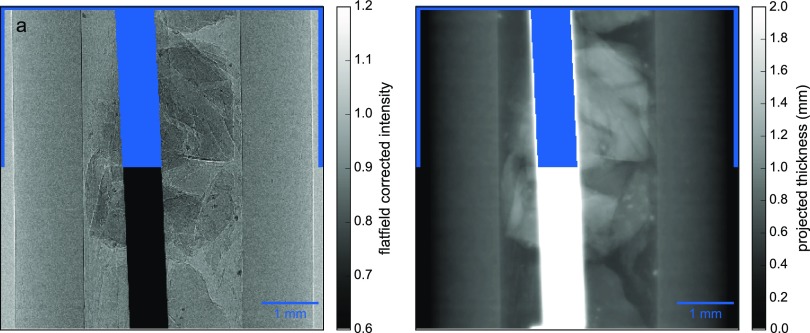
Intensity and trace of the first projection. In (a), the flat-field corrected intensity for the first angle is shown. The associated recovered trace using PAG is depicted in (b). In both images, the identical mask, which completely covers the tungsten wire on the projection during the reconstruction, is shown only on the upper half of the images.

In the following, we demonstrate some benefits of our fully iterative algorithm STIR compared to previous implementations, such as combinations of PAG with FBP or PAG together with SIR. The sample consists of a perfusion tube made of polyethylene (PE) as well as acrylic glass (PMMA) spheres that have been crushed with pliers. In addition, a thread made of tungsten is added. This example is motivated by limitations of the discussed imaging techniques when the single material assumption is not met. This includes for instance resolving soft-tissue features in the vicinity of bone structures. The projections are obtained at an effective propagation distance of 28 mm with a source-to-sample distance of 45 mm and a sample-to-detector distance of 74 mm. The effective voxel size corresponds to 2.56 *μ*m. In Fig. 3(a), the flat-field corrected intensity at the detector plane for the first angle is depicted. As anticipated, PE and PMMA have similar absorption and phase-shifting properties. In contrast, the tungsten thread is highly absorbing.

Figure 3(b) shows the trace of Fig. 3(a) recovered by PAG. The parameters for *δ* and *μ* are chosen for PMMA. Therefore, PE and PMMA are accurately recovered. The difficulties in whole-sample reconstruction arise from the trace of the highly absorbing tungsten where the assumed absorption and phase shifting properties are not met. This means that the trace of the tungsten is smeared out, covering features located in the vicinity that are lost from now on, which results in a significant drawback for two-step reconstruction techniques.

**FIG. 4.**
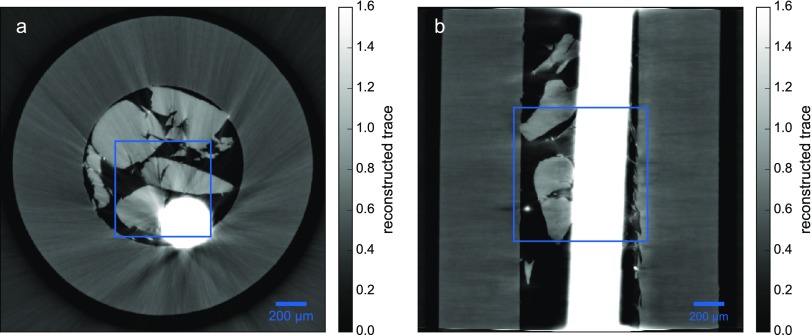
FBP reconstruction of the traces recovered by PAG. (a) shows a transverse and (b) a longitudinal slice of the sample shown in projection in Fig. 3. The positions of the enlarged views in Fig. 5 are depicted by the blue squares.

A transverse and a longitudinal slice recovered by the FBP acting on the projected thicknesses recovered by PAG are depicted in Fig. 4. The areas where tungsten is present have large values and yield severe artifacts. Moreover, these areas are smeared out covering features in their vicinity.

To overcome the artifacts introduced in the analytical reconstruction approach, one can make use of the additional weights $w_{i}^{\theta }$ in iterative reconstruction techniques to mask out regions on the projections where the homogeneity assumption is violated, namely, in regions where the tungsten thread is present. The calculation of these weights is discussed in Sec. VII. The upper parts of Fig. 3 illustrate the position of the mask noting that due to the smearing of the PAG algorithm, it does not cover the artifacts on the traces entirely. These weights are applied according to Eq. (15) for STIR and Eq. (17) for SIR.

Additionally total-variation regularization is used according to Eq. (18). The strength *λ* of the regularizer is chosen in a way to make the noise level for both iterative reconstruction techniques compatible. Due to the variability of the artifacts, the parameters are chosen empirically rather than by defining regions, where well-defined noise characteristics have to be fulfilled. The volumes to reconstruct are initialized with zeros in the iterative approaches to make the outcome independent of the starting conditions. This however increases the number of iterations significantly until a stable state is found. Thereby, 100 and 800 L-BFGS iterations are chosen for SIR and STIR, respectively, ensuring that a stable state is reached.

**FIG. 5.**
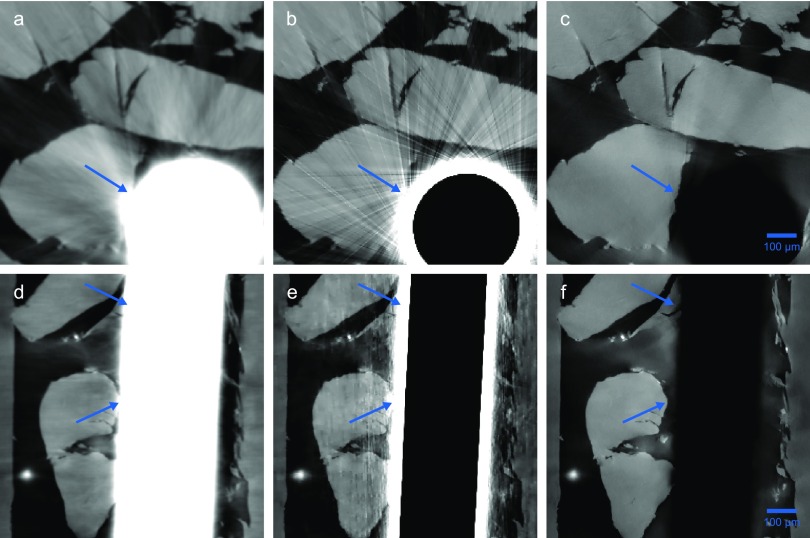
Transverse and longitudinal slices for different reconstruction techniques. The positions of the enlarged views are depicted in Fig. 4 for the transverse and longitudinal slice with an equal grayscale. The enlarged views of the reference reconstruction obtained by PAG + FBP are depicted in (a) and (d). The results of the iterative reconstruction algorithms depicted in (b) and (e) for PAG + SIR and (c) and (f) for STIR make use of additional weights as illustrated in the upper parts of Fig. 3 and regularization techniques.

To illustrate the effects of the different reconstruction techniques, the regions marked in Fig. 4 are enlarged for the three approaches in Fig. 5. Consequently, Figs. 5(a) and 5(d) show the same content as in Figs. 4(a) and 4(b), respectively. The results obtained by PAG with SIR can be seen in Figs. 5(b) and 5(e). Finally, recovering the three-dimensional distribution of our sample by the fully iterative approach STIR yields the results depicted in Figs. 5(c) and 5(f).

The following comparison refers to the transverse as well as longitudinal slices. The mean values of the PMMA spheres do not change significantly over the different reconstruction techniques, making the iterative approaches as quantitative as the conventional approach. The main differences between the various reconstruction algorithms arise from the tungsten thread. As mentioned before, the FBP reconstruction of the traces leads to the fact that the tungsten thread is smeared out, rendering the features in the vicinity useless and resulting in streak-like artifacts throughout the whole image. Replacing the FBP with a SIR reconstruction, the areas corresponding to regions that are masked out on the traces contain hardly any signal in the reconstructed volume. However, due to the smearing of the PAG algorithm, the areas in the vicinity of the tungsten thread cannot be correctly recovered and artifacts arise from these regions. Finally, the one-step STIR approach can circumvent these problems by masking out the tungsten thread entirely on the projections by using the same mask as before. Consequently, features in the vicinity are recovered and the volume does not suffer from severe streak artifacts. Furthermore, while the FBP reconstruction cannot make use of any regularization techniques, the edges of the sample are not as sharp and the noise level is higher than in the iterative approaches that incorporate total-variation regularization. Unfortunately, the edge-preserving properties of this regularization technique emphasize the streak artifacts of the SIR method. However, the STIR method most strongly benefits from the noise reducing and edge preserving capability of the regularization. In conclusion, although the STIR method provides improved image quality and is able to recover features in the vicinity of highly absorbing objects, there are still small residual streak artifacts arising from the tungsten thread leaving space for further improvements. The supplementary material provides a difference map of the transverse slice between the FBP and STIR reconstruction and discusses the differences in more detail.

## VI. CONCLUSION

We validated the approximations of our approach before comparing the proposed algorithm STIR with widely used two-step approaches, namely, recovering the traces with PAG before recovering the spatial distribution of the material by FBP approaches or SIR techniques for tomography. We find that our approach allows for improved reconstruction of features in the vicinity of highly absorbing objects compared to two-step approaches. Furthermore, STIR is well suited as a refinement step to the conventional reconstruction approach to improve the image quality with statistical modeling, no intermediate phase-retrieval, and the use of regularization techniques.

As our results focus on the validation of our reconstruction techniques compared to well-known approaches, we omit a detailed evaluation of the statistical nature of our algorithms in order not to bias our comparisons and hence only evaluate the benefits of using binary weights to emphasize the limitations of PAG + SIR compared to STIR. In principle, our one-step approach is capable of handling the noise properties directly from the projections with arbitrary noise models, which is a huge benefit compared to two-step approaches, where for an iterative tomographic reconstruction, the statistical weights have to be applied on the traces.

With the recent advances in x-ray sources including liquid-metal jet tubes and compact synchrotrons, propagation-based PCI is increasingly transferred from synchrotron facilities to laboratories. With the comparably small flux and high noise levels of laboratory sources, our statistical approach has the potential of improving image quality significantly, where a correct modeling of noise is crucial. Furthermore, our versatile forward model can easily be extended to include, for instance, source and detector models directly into our reconstruction framework.

As a remark, although implicitly assumed in the derivation, the requirement for monochromatic x-rays is not particularly strict for this imaging technique as a change in energy only slightly alters the fringes. In our case, we use a commercially available polychromatic laboratory source to validate our reconstruction algorithms. Moreover, our approach can be applied to more strongly absorbing objects without any modifications by changing the corresponding values for the complex refractive index.

Although successfully handling highly absorbing objects, our algorithm still imposes the homogeneity assumption for the remaining materials. Recent multi-distance algorithms successfully implemented at synchrotron facilities can relax this assumption to allow for the reconstruction of heterogeneous objects.^16^ However, our approach is designed for single-distance methods with laboratory sources in mind, where the statistical nature of our algorithm benefits the most. Future work could investigate how our model can be extended to allow for more than one material, using, for instance, concepts used in iterative multi-distance algorithms, for example, sophisticated regularization techniques.

One drawback is the computational cost of STIR. This cost is not necessarily a consequence of the more sophisticated forward model compared to absorption CT. The projection operations remain the most time-consuming operations that are also present in SIR, and the additional overhead of the operations related to the propagation of the x-rays, namely, the Laplacian operations, is small. For now, adapting the solver to minimize our non-linear model in fewer iterations and making more explicit statements about the optimization problem is a challenge that could be addressed in the future. However, the number of iterations relies heavily on the initial guess of the iterative algorithms. For instance, for absorption CT, one would use the FBP as the start image; consequently, PAG + FBP would be a decent start image for propagation-based phase-contrast CT by STIR and would significantly improve the speed of convergence. In order not to bias the comparison between the different reconstruction techniques by such an initial guess, we accepted the increase in computational time to show that our algorithms converge, regardless of the initial guess, even from an array of zeros.

With the availability of more powerful computers, we believe that our approach can prove to be beneficial for propagation-based phase contrast CT in the fields of medicine, biology, and manufacturing, using x-rays, visible light, electrons, or neutrons, in particular, for applications with low flux and high noise levels or when there are spatially close sample materials with quite different optical properties. Our approach should work for all applications that until now rely on the PAG algorithm for phase retrieval as for instance imaging of bones, lungs, and brains in biology or examining cracks or defects in materials science.

## VII. METHODS

The authors state that an ethics approval is not required.

### A. Calculation of the statistical weights

The statistical weights reflect the reliability of our model. In the presented tomographic case, it is violated for the tungsten thread. Thereby, the tungsten thread is selected by thresholding the flat-field corrected intensity at values lower than 0.5. Afterwards, this mask is slightly dilated in order to cover the areas of the edge enhancement. Furthermore, to avoid effects at the borders, regions within 15 pixels of the borders are also masked. The statistical weights are set to zero within the masked areas. Finally, in order not to bias the comparison of the different reconstruction techniques to their statistical properties, additional statistical weighting of the measurements was omitted by setting weights outside of the masked area to one.

## VIII. SUPPLEMENTARY MATERIAL

See supplementary material for the difference map of the proposed algorithm STIR depicted in Fig. 5(a) and the conventional approach of applying a FBP on the traces recovered by PAG of Fig. 5(c).
